# Differences in adiposity trajectories by birth cohort and childhood social class: evidence from cohorts born in the 1930s, 1950s and 1970s in the west of Scotland

**DOI:** 10.1136/jech-2013-203551

**Published:** 2014-02-06

**Authors:** Richard J Shaw, Michael J Green, Frank Popham, Michaela Benzeval

**Affiliations:** 1 MRC/CSO Social and Public Health Sciences Unit, University of Glasgow, Glasgow, UK; 2 Institute for Social and Economic Research, University of Essex, Colchester, UK

**Keywords:** Cohort studies, Longitudinal Studies, Obesity, Social Inequalities

## Abstract

**Background:**

Since the 1930s, the environment has become increasingly obesogenic, leading to rising rates of adiposity and socioeconomic inequalities in adiposity. Building on studies comparing body mass index (BMI) for cohorts born over a period of 20 years, we examine the social patterning of BMI and central adiposity for three cohorts born over a 40-year period.

**Methods:**

Using data from the West of Scotland Twenty-07 study (n=4510), we investigate 20-year trajectories of adiposity for three cohorts born in the 1930s, 1950s and 1970s, allowing us to study 60 years of the lifecourse. Stratified by gender, we employed multilevel models to generate trajectories for BMI and waist-to-height ratio (WHtR) and explored how these trajectories varied by childhood social class.

**Results:**

Adiposity increased most quickly with age in the youngest cohort, and cohort differences were greater than socioeconomic differences. For example, the smallest cohort difference for BMI, a comparison of men in the 1930s and 1950s cohorts at age 55, was 2.66 (95% CI 2.11 to 3.20) kg/m^2^, while the largest socioeconomic difference, a comparison of manual and non-manual women at age 64, was 1.18 (95% CI 0.37 to 1.98) kg/m^2^. Socioeconomic inequalities in adiposity increased with age and were greater for women than for men. The results for WHtR differed in that increases in WHtR accelerated with age while increases in BMI slowed.

**Conclusions:**

Socioeconomic differences in adiposity accumulate slowly across the lifecourse and are approximately only a third of the adiposity differences between cohorts.

## Introduction

In the UK in the 1930s, undernutrition was a greater challenge than obesity and it was those wealthy not those poor who had the greatest obesity risk.^1^ Subsequent technological and societal changes have increased exposure to obesogenic factors in the environment.^2^ The consequences of these changes include high rates of obesity^2^ and the relationship between socioeconomic position (SEP) and adiposity changing from a positive to a negative one.^3^ Cohort comparison studies indicate that body mass index (BMI) rises faster in more recently born cohorts.^4–7^ However, these studies either have relatively short follow-up periods,^6^ do not investigate cohort variations in the influence of SEP,^4^ compare cohorts born within the same 20-year period^4^ ^5^ ^7^ or have data spanning less than 30 years of the lifecourse.^5^ ^7^ In this paper, we explore how adiposity trajectories spanning 60 years of the lifecourse vary by cohort and SEP for the critical period of childhood.

Childhood SEP has been associated with adult adiposity independently of adult SEP.^7–11^ Disadvantaged childhood SEP may lead to increased vulnerability to adult adiposity because childhood is a critical period for the determination of adult diet,^12^ physical activity^13^ and emotional well-being;^14^ ^15^ however, the extent to which this vulnerability is translated into adiposity may be dependent on obesogenic conditions in the environment, with socioeconomic difference becoming apparent at an earlier age in more recently born cohorts.^10^ ^16^ ^17^ Consequently, cohort variations in degree of exposure to an obesogenic environment may influence the patterning of adult adiposity trajectories by childhood SEP.

A lifecourse perspective is needed when measuring adiposity. BMI, the most commonly used measure of adiposity, is an indicator of bone and muscle mass.^18^ Increases in adiposity may be masked by corresponding falls in other tissue due to chronic disease and sarcopenia.^19^ At older ages measures of central adiposity, such as waist-to-height ratio (WHtR), are better indicators of disease and mortality risk.^20^ ^21^ A longitudinal study from the ages of 36–53^11^ and a cross-sectional comparison of two cohorts, one aged 43 and the other 45,^4^ would suggest that socioeconomic inequalities in adiposity appear to be similar whether adiposity is measured using BMI or central adiposity.^4^ ^11^ However, this may not hold for trajectories of older adults.

In this study, we use data from the West of Scotland Twenty-07 study to investigate how adiposity trajectories spanning 60 years of the lifecourse differ between three cohorts each born 20 years apart. We also explore how these trajectories vary by childhood SEP. In addition, we investigate whether we draw the same conclusions if we measure adiposity using WHtR rather than BMI.

## Methods

### Study population

The Twenty-07 study has followed up people in three cohorts born around 1932, 1952 and 1972 for 20 years (for full details, see Benzeval *et al*^22^). The study contains two subsamples: the regional sample, a two-stage stratified random sample of people living in the Central Clydeside Conurbation, west of Scotland and the localities sample of people from two areas in the city of Glasgow. Baseline interviews were carried out in 1987/1988 when study members were aged approximately 15, 35 and 55. The target sample for each cohort was 1500. The overall achieved sample was 4510, with 1515 in the 1970s cohort, 1444 for the 1950s cohort and 1551 for the 1930s cohort. This represents 85% of those approached for interview for the 1970s cohort, 89% for the 1950s and 87% for the 1930s. At baseline, study members have been shown to be representative of the general population in the sample area.^23^ The study members were followed up on separate occasions with 86.1% of those alive responding in 1990/1992, 68.8% in 1995/1997, 64.3% in 2000/2004 and 67.4% 2007/2008. By wave 5, mortality had accounted for 1.7% of the 1970s cohort, 6.0% of the 1950s cohort and 36.6% of participants in the 1930s cohort.^24^ Numbers participating in each wave and percentage missing for each variable by cohort and gender are presented in the online supplementary table.

### Measures

Height, weight and waist circumference were recorded by nurses at the baseline interview and subsequent waves, with the exception of the localities sample who self-reported measures via a postal questionnaire for wave 3. BMI was calculated by dividing weight by height squared. WHtR was calculated by dividing waist by height. Women were coded as missing for waves in which they were pregnant.

Childhood SEP was assessed using Registrar General's 1980 classification,^25^ dichotomised into non-manual and manual, for the head of household's occupation when participants were aged 15. In households with two parents, the father was assumed to be the head of household. If he did not have an occupation, then the mother's occupation was used instead. For the 1970s cohort, participants’ parents were directly asked what their current or most recent occupation was at baseline data collection. For the 1950s and 1930s cohorts, participants were asked to recall what their parents’ main occupation was at age 15. There were slight differences in the way the question was asked between cohorts, but nevertheless we were able to code into occupational classes effectively. We discuss the potential impact of these differences below.

### Statistical analyses

Analyses were conducted using multilevel models with measurement occasions, nested within individuals, nested within geographic sampling units. Multilevel models take account of the clustered nature of data and enable cases to be used for all occasions on which individuals have data available. The models are potentially unbiased assuming the data in the model are missing at random. Hot deck imputation by cohort and sample type was used to impute age for seven observations for which the questionnaire completion date was missing.

We created three models each for both BMI and WHtR, stratified by gender. In the base model, we included fixed effects for manual social class, dummy variables for cohort (1970s cohort used as reference) and linear and quadratic terms for age. The quadratic term was included in order to account for a non-linear relationship between age and adiposity. In addition, we included random intercepts for each individual and sampling unit and a random slope for age. In model 2, we added interaction terms between cohort and the linear and quadratic terms for age in order to test whether the trajectories varied by cohort. In model 3, we added interactions terms between cohort, social class and age in order to investigate how the effects of SEP varied by cohort. Likelihood ratio tests were used to investigate whether the additional variables improved model fit.

All models were estimated using the xtmixed command in Stata 12.1 using maximum likelihood estimation. Model fit assumptions were assessed by plotting the residuals and random effects against a normal distribution, the assumption of normality held at the occasion and the individual levels. In all models, age was centred at 45 to reduce collinearity and to ensure that the intercepts were for a time point covered by the range of the data. For the results presented in the tables, WHtR was multiplied by 100 to make interpretation of coefficients easier.

## Results

Descriptive statistics for study participants are shown in table 1. BMI increased with each wave with the greatest increases in the younger cohorts. There was also a tendency for the proportion of people from a manual background to fall in each wave.

**Table 1:** Descriptive statistics for the main outcomes and explanatory variables for the three cohorts in the Twenty-07 study

	Baseline	Wave 2	Wave 3	Wave 4	Wave 5
	1987/1988	1990/1992	1995/1997	2000/2004	2007/2008
*Men*					
1970s cohort					
Number in cohort at each wave	737	637	419	384	424
Average age (years)	15.7	18.6	24.8	30.1	36.7
Mean BMI (kg/m^2^)	20.3	22.5	24.7	26.3	27.9
Mean WHtR	0.44	0.45	0.48	0.51	0.55
% of cohort manual childhood social class	39.5	36.4	35.2	33.6	35.2
1950s cohort					
Number in cohort at each wave	656	549	456	446	457
Average age (years)	36.2	40.5	45.3	50.2	57.1
Mean BMI (kg/m^2^)	25.1	26.0	26.4	27.7	28.6
Mean WHtR	0.51	0.52	0.53	0.55	0.58
% of cohort manual childhood social class	70.91	71.2	70.4	68.9	67.8
1930s cohort					
Number in cohort at each wave	702	580	450	368	279
Average age (years)	56.2	59.6	64.3	69.1	76.1
Mean BMI (kg/m^2^)	25.9	26.2	26.7	27.2	27.3
Mean WHtR	0.55	0.55	0.56	0.57	0.59
% of cohort manual childhood social class	84.4	83.8	81.4	80.4	80.4
*Women*					
1970s cohort					
Number in cohort at each wave	778	705	496	459	518
Average age (years)	15.8	18.6	24.9	30.2	36.7
Mean BMI (kg/m^2^)	21.1	22.8	24.3	26.3	27.6
Mean WHtR	0.43	0.44	0.46	0.49	0.54
% of cohort manual childhood social class	40.7	38.6	34.4	34.5	35.0
1950s cohort					
Number in cohort at each wave	788	676	570	534	542
Average age (years)	36.2	40.5	45.2	50.1	57.0
Mean BMI (kg/m^2^)	24.3	25.5	26.4	27.6	28.4
Mean WHtR	0.47	0.49	0.50	0.53	0.58
% of cohort manual childhood social class	68.4	67.6	66.5	64.5	64.8
1930s cohort					
Number in cohort at each wave	849	686	580	470	384
Average age (years)	56.3	59.7	64.5	69.2	76.3
Mean BMI (kg/m^2^)	26.0	26.5	26.9	27.6	27.9
Mean WHtR	0.51	0.52	0.53	0.55	0.59
% of cohort manual childhood social class	77.5	76.9	75.1	74.3	72.3

BMI, body mass index; WHtR, waist-to-height ratio.

**Figure 1:**
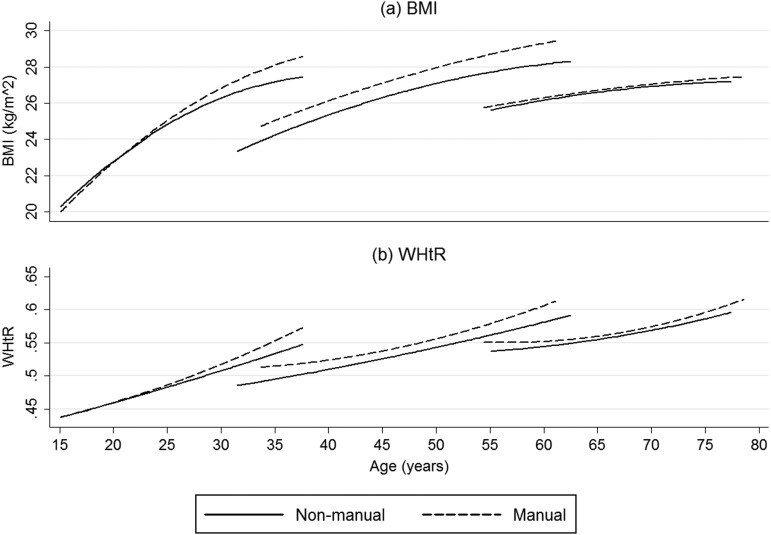
Estimated body mass index and waist-to-height trajectories for men in the 1970s,1950s and 1930s cohorts in the West of Scotland Twenty-07 study, 1987–2008.

The multilevel models predicting BMI by cohort for men are presented in table 2. Model 2 demonstrates that BMI trajectories vary by cohort, while model 3 demonstrates that there was a three-way interaction between social class, age and cohort, which is presented in figure 1A. When cohorts are compared at the start of the study, the more recently born cohorts had lower BMIs, but BMI increases are also greater for more recently born cohorts so that when cohorts were compared at the same age the more recently born cohort had a higher BMI. Using model 2 the difference in predicted BMI at age 35 between the 1970s and 1950s cohorts was 2.79 (95% CI 2.37 to 3.20) kg/m^2^ and the difference at age 55 between the 1930s and 1950s cohorts was 2.66 (95% CI 2.11 to 3.20) kg/m^2^. Model 3 investigated whether there were cohort differences in how BMI trajectories varied by childhood social class. Social class differences were significant but small in magnitude compared with the cohort differences, with the largest socioeconomic difference between manual and non-manual workers being only 0.99 (for the 1950s cohort at age 55; 95% CI 0.16 to 1.82) kg/m^2^. Social class inequalities differed by cohort, and for men in the 1930s cohort there was little evidence of a socioeconomic gradient. Where there were socioeconomic differences in BMI, these would appear to be the consequence of gradual processes that increases with age. For the 1970s cohort, the difference in BMI was small initially but increases to 0.92 (95% CI 0.16 to 1.82) kg/m^2^ by age 35. For the 1950s cohort, at age 35 there was a difference of 0.81 (95% CI 0.19 to 1.42) kg/m^2^ and this continues to increase with age up to 0.99 (95% CI 0.16 to 1.82) kg/m^2^ at age 55.

**Table 2:** Regression coefficients from multilevel models for expected BMI in kg/m^2^ and WHtR for men in the three cohorts of the West of Scotland Twenty-07 study, 1987–2008

	BMI†			WHTR‡		
	Model 1	Model 2	Model 3	Model 1	Model 2	Model 3
Main effects						
Age	0.204***	−0.066*	−0.098**	0.376***	0.696***	0.573***
Age^2^	−0.004***	−0.011***	−0.011***	−0.003***	0.004**	0.002
Manual social class	0.273	0.283	1.821***	0.781***	0.778**	4.900
1950s cohort	−2.904***	−1.148***	−1.003***	−3.640***	−7.395***	−6.298***
1930s cohort	−6.285***	−3.504***	−3.177***	−7.384***	−4.655***	−5.574***
*Interactions*						
Cohort×age						
1950s×age		0.246***	0.272***		−0.371***	−0.240***
1950s×age^2^		0.008***	0.008***		0.003	0.000
1930s×age		0.205***	0.270***		−0.961***	−0.602***
1930s×age^2^		0.009***	0.009***		0.008***	0.004+
Social class×age						
Manual×age			0.100**			0.364***
Manual×age^2^			0.001			0.007***
Social class×age×cohort						
Manual×1950			−1.020+			−3.722***
Manual×age×1950s			−0.091*			−0.375***
Manual×1930			−1.307*			−1.302
Manual×age×1930s			−0.139+			−0.654
Constant	29.902***	27.823***	27.312***	57.277***	60.264***	58.877***
Random effects						
Random intercept area	5.92×10^−7^	1.91×10^−6^	2.80×10^−6^	2.38×10^−6^	1.86×10^−5^	2.66×10^−6^
Random intercept person	3.783	3.787	3.765	5.005	5.006	4.984
Random slope age	0.106	0.106	0.104	0.122	0.125	0.122
Correlation intercept slope	0.642	0.641	0.639	0.692	0.683	0.686
Residual	1.443	1.421	1.423	3.039	2.978	2.977
Likelihood	−15 870.553	−15 806.243	−15 792.049	−20 009.702	−19 922.066	−19 901.003
χ^2^ (df)		128.62 (4)	28.39 (6)		175.27 (4)	42.13(6)
p Value for χ^2^		<0.0001	<0.0001		<0.0001	<0.0001

+p<0.10, *p<0.05, **p<0.01, ***p<0.001.

†Measurement occasions N=6951, individuals N=1943, areas N=62.

‡Measurement occasions N=6982, individuals N=1946, areas N=62.

BMI, body mass index, WHtR, waist-to-height ratio.

The results for WHtR by cohort for men are also shown in table 2. They are very similar to those for BMI. Model 2 demonstrates that WHtR trajectories vary by cohort, and there was a three-way interaction between social class, cohort and age, which is presented in figure 1B. The main difference between the WHtR and BMI trajectories relates to how the trajectory gradients vary with increasing age. In the BMI models, the main quadratic terms were negative, indicating that the increases in BMI slow with advancing age. In contrast, in model 3 for WHtR, the main quadratic term and the quadratic term for manual social class were positive, indicating that increases in WHtR accelerate with age, particularly for those from manual backgrounds.

**Figure 2:**
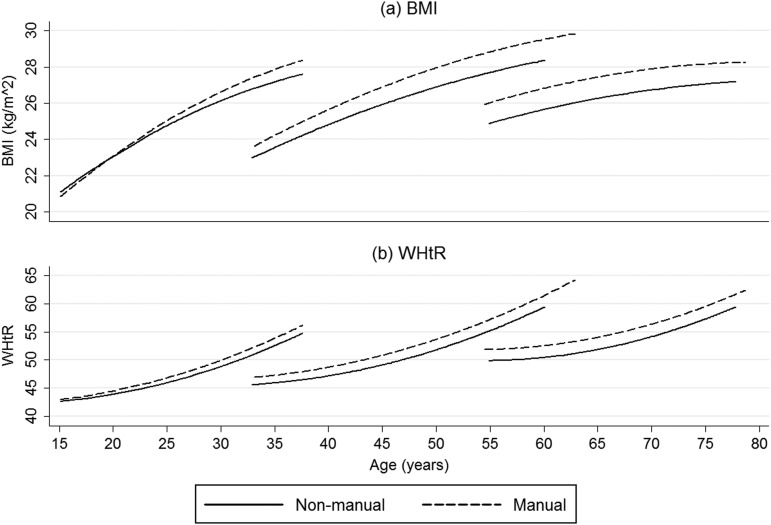
Estimated body mass index and waist-to-height trajectories for women in the 1970s,1950s and 1930s cohorts in the West of Scotland Twenty-07 study, 1987–2008.

For women, the models predicting BMI and WHtR are presented in table 3, with the final models being presented in figure 2. The trajectories were similar to those for men, with the cohort differences greater than the socioeconomic differences. However, there were differences between the genders. In model 2 cohort, differences in adiposity for women were greater than cohort differences in adiposity for men. For women, the difference in BMI at age 35 between the 1970s and 1950s cohorts was 3.52 (95% CI 3.02 to 4.03) kg/m^2^ and the difference at age 55 between the 1930s and 1950s cohorts was 2.73 (95% CI 2.04 to 3.41) kg/m^2^. In addition, for BMI and WHtR there was no evidence that the associations with SEP varied by cohort, so three-way interaction terms were omitted from model 3 and the influence of social class on the trajectories appears to be the same for women in all cohorts. The difference in BMI between women from manual and non-manual backgrounds increased with age until a maximum difference of 1.18 (95% CI 0.37 to 1.98) kg/m^2^ at the age of 64, and decreases thereafter. For WHtR, inequalities increased for the whole period for which we have data.

**Table 3:** Regression coefficients from multilevel models for expected BMI in kg/m^2^ and WHtR for women in the three cohorts of the West of Scotland Twenty-07 study, 1987–2008

	BMI†			WHTR‡		
	Model 1	Model 2	Model 3	Model 1	Model 2	Model 3
Main effects						
Age	0.215***	0.055+	0.048	0.493***	1.158***	1.147***
Age^2^	−0.003***	−0.007***	−0.006***	−0.001**	0.016***	0.016***
Manual social class	0.385+	0.388+	0.961***	0.998***	0.964**	1.709***
1950s cohort	−3.721***	−2.171***	−7.827***	−6.874***	−12.984***	−13.246***
1930s cohort	−6.336***	−5.076***	−5.364***	−11.971***	−10.704***	−11.055***
*Interactions*						
Cohort×age						
1950s×age		0.167***	0.160***		−0.672***	−0.682***
1950s×age^2^		0.003**	0.003**		−0.002	−0.002
1930s×age		0.183***	0.175***		−1.452***	−1.464***
1930s×age^2^		0.003**	0.004**		0.001	0.001
Social class×age						
Manual×age			0.023*			0.03*
Manual×age^2^			−0.001*			−0.000
Constant	29.928***	28.496***	28.314***	56.980***	62.605***	62.363***
Random effects						
Random intercept area	0.413	0.417	0.400	0.955	0.930	0.927
Random intercept person	5.274	5.274	5.271	6.828	6.886	6.885
Random slope age	0.150	0.150	0.149	0.174	0.180	0.179
Correlation intercept slope	0.732	0.730	0.732	0.810	0.802	0.803
Residual	1.748	1.742	1.742	3.497	3.370	3.370
Likelihood	−21 066.572	−21 053.371	−21 045.915	−25 399.418	−25 219.131	−25 214.27
χ^2^ (df)		26.40 (4)	14.91 (2)		360.57	9.72
p Value forχ^2^		<0.0001	0.0006		<0.0001	0.0077

*p<0.05, **p<0.01, ***p<0.001.

†Measurement occasions N=8417, individuals N=2245, areas N=62.

‡Measurement occasions N=8347, individuals N=2244, areas N=62.

BMI, body mass index, WHtR, waist-to-height ratio.

## Discussion

We use data from three cohorts each born 20 years apart to create trajectories of adiposity spanning 60 years of the lifecourse. These trajectories vary by cohort and childhood SEP, and the associations between cohort and adiposity are greater than the associations between childhood SEP and adiposity. While the trajectories for BMI and WHtR are very similar, there are important differences, which will be addressed below.

The broad pattern of BMI, increasing with age but with the rate of increase decelerating as age increases and a faster rate of increase in younger cohorts, is generally consistent with other studies.^4–7^ ^26^ The cohort differences in adiposity in our study are greater than those found in other cohort comparisons. The smallest cohort difference in our study, a comparison of men in the 1950s and 1930s cohorts at age 55, is 2.66 kg/m^2^. In contrast, a study comparing the UK 1946 cohort at age 43 with the 1958 cohort at age 45 found a 1.8 kg/m^2^ difference for men and 1.2 kg/m^2^ for women.^4^ While a comparison of cohorts born 30 years apart in the Netherlands found that at any given age there was a BMI difference of 2.1 kg/m^2^.^6^

We found associations between childhood SEP and adiposity in five out of six cohort and gender combinations. For both genders, in the 1970s and 1950s cohorts social inequalities in adiposity increased with age; however, members of the 1970s cohort were nearly 30 before a significant socioeconomic difference emerged. There were no inequalities for men in the 1930s cohort, while for women inequalities increased until the age of 64. The subsequent narrowing for women in the 1930s cohort requires cautious interpretation because this result was not repeated for WHtR and may indicate measurement issues (see below). The increase in inequalities until retirement age would suggest that inequalities in adiposity develop through cumulative processes.

While we find little evidence of cohort variations in socioeconomic inequalities in adiposity for women, for men, where ages were comparable, there were greater socioeconomic inequalities in more recently born cohorts. This is partly consistent with the concept of socioeconomic differences in adiposity being greater in cohorts who have developed in a more obesogenic environment. In the UK 1958 birth cohort, social class differences had emerged by the age of 23,^10^ which is slightly younger than our results for the 1970s cohort. The older age in our study may be a consequence of the relatively small numbers of non-manual class people in the 1970s cohort and/or the effect of a different environment in Scotland.

In general, the trajectories for BMI and WHtR are similar. However, we do find some differences between the measures. Increases in BMI decelerate with age, suggesting that levels of obesity may stabilise. In contrast, increases in WHtR accelerate with age. A similar concern is raised by changes in socioeconomic inequalities in adiposity for women in the 1930s cohort. The BMI model indicates that inequalities narrow after the age 64, while the WHtR model indicates that inequalities continue to increase until the age of 75. It is possible that when BMI is used to indicate adiposity that increases in fat mass are being masked by changes in lean tissue and consequently BMI may underestimate inequalities in older people.

Gender had an important influence on the results of our study. Consistent with the literature more broadly,^8^ ^27^ cohort and socioeconomic differences in adiposity are larger for women than for men. This is most dramatically illustrated in the 1930s cohort. For women, the socioeconomic inequalities were greatest in this cohort, indicating that adiposity may be the consequence of accumulative effects across the lifecourse. In contrast, for men in the 1930s cohort, there was little evidence of any association between childhood SEP and adiposity. Given that men have a shorter life expectancy, one possible explanation is that interactions between adiposity, SEP and mortality could have led to survival biases^24^ or greater reductions in fat-free mass for men from disadvantaged backgrounds, resulting in a narrowing of adiposity inequalities with age for men. However, rather than a narrowing of inequalities, we find a lack of evidence for any socioeconomic gradient in adiposity for men in this cohort, which may reflect the childhood circumstances of this cohort and gender differences in susceptibility to subsequent environmental changes.

The 1930s cohort were born into the great depression when undernutrition was a greater challenge than obesity,^1^ following this between 1939 and 1954 rationing and food controls largely eliminated socioeconomic inequalities in diet.^28^ Thus an absence of adiposity socioinequalites for men in this cohort is perhaps unsurprising. However, this raises the question of why socioinequalities in adiposity subsequently develop for women in this cohort but not men? Answering this question will be difficult. A person's adiposity is a consequence of a complex system, and changes in adiposity trajectories are likely to be influenced by factors from numerous domains.^29^ Some factors may have altered the influence of social class on health generally, for example, declining work-related physical activity over the last 50 years.^30^ Other factors may be targeted at specific groups, for example, advertisements for sugary and fatty foods are more common in magazines targeted at women from disadvantaged backgrounds.^31^

A strength of the study is that all three cohorts are from the same area and time period, with consistent collection methodology. While it is logically impossible to distinguish between cohort and period effects,^32^ we believe that the population from which the cohorts are drawn is reasonably stable. Therefore, we believe that cohort variations in adiposity trajectories can be attributed to cohort-specific interactions between age and environment. However, the homogenous nature of the population may limit generalisability to some populations.^23^ Increased rates of childhood obesity and socioeconomic inequalities in obesity^33^ may suggest that more recently born cohorts, and especially children with a disadvantaged SEP, are likely to be on adiposity trajectories steeper than those found for the 1970s cohort.

The data, spanning 15–75 years, allow us to explore 60 years of the lifecourse; however, overlap for adiposity data is restricted to the first and final waves and thus relatively limited. Inspection of residuals suggested all the models were a good fit for the data for the 1930s and 1950s cohorts; the BMI models for the 1970s cohort may be slightly too simple to fully capture late adolescent growth in the first three waves. Additionally there were differences in the way the SEP data were collected and slightly different wording between cohorts, all of which may lead to a slight bias towards reporting more advantageous childhood circumstances in the older cohorts.^34^

Overall the study has good response rates for each wave. However, the assumption that the data are missing at random is contingent only on adiposity, and socioeconomic data and other potential biases, particularly survival, may not have been accounted for.

The social and environmental changes over the last 80 years have had a dramatic impact on levels of adiposity. Our results indicate that adiposity has increased in more recently born cohorts and that social inequalities increase with age, and for men, in more recently born cohorts. Overall the impact of birth cohort is greater than that of childhood SEP. If cohort differences observed here continue, levels of obesity will become an even bigger public health challenge in the future.

**What is already known on this subject**

- Body mass index (BMI) increases more rapidly in more recently born cohorts.
- The relationship between socioeconomic position and adiposity has changed in more recent decades.
- Waist-to-height ratio and BMI trajectories appear similar up to midlife, but theory suggests that this may not be true for older ages.

**What this study adds**

- For women, the association between social class and adiposity appears to be the same across cohorts. However, for men at comparable ages, socioeconomic inequalities in adiposity may be greater in more recently born cohorts.
- In the west of Scotland, socioeconomic differences in adiposity are approximately only a third of the adiposity differences between cohorts.

## Supplementary Material

Web supplement
