# Supplementary material for: Differences in adiposity trajectories by birth cohort and childhood social class: evidence from cohorts born in the 1930s, 1950s and 1970s in the west of Scotland
Source: J Epidemiol Community Health. 2014 Feb 6;68(6):550–6. doi: 10.1136/jech-2013-203551 (PMC4033148; doi:10.1136/jech-2013-203551)
Supplement: Web supplement [file jech-2013-203551-s1.pdf]

Supplementary table. Number of people participating in each wave and percentage of those participating with missing data for each variable by cohort and gender.

	Baseline 1987/8	Wave2 1990/2	Wave3 1995/7	Wave4 2000/4	Wave5 2007/8
Men					
**1970s Cohort**					
Number in cohort at each wave	737	637	419	384	424
% Missing body mass index	5.0	2.0	4.1	3.1	1.4
% Missing waist to height ratio	4.6	1.9	3.6	1.3	2.8
% Missing childhood social class	1.2				
**1950s Cohort**					
Number in cohort at each wave	656	549	456	446	457
% Missing body mass index	7.3	0.9	7.9	3.8	1.1
% Missing waist to height ratio	7.0	1.3	6.8	0.9	1.8
% Missing childhood social class	4.0				
**1930s Cohort**					
Number in cohort at each wave	702	580	450	368	279
% Missing body mass index	6.7	0.7	8.7	6.3	6.8
% Missing waist to height ratio	6.4	1.0	7.8	4.6	6.8
% Missing childhood social class	6.8				
Women					
**1970s Cohort**					
Number in cohort at each wave	778	705	496	459	518
% Missing body mass index	6.2	0.9	1.2	4.6	4.5
% Missing waist to height ratio	5.9	2.4	3.2	10.7	8.1
% Missing childhood social class	2.2				
**1950s Cohort**					
Number in cohort at each wave	788	676	570	534	542
% Missing body mass index	5.7	0.74	1.2	3.4	2.6
% Missing waist to height ratio	7.0	1.8	1.2	3.2	3.1
% Missing childhood social class	4.6				
**1930s Cohort**					
Number in cohort at each wave	849	686	580	470	384
% Missing body mass index	6.1	1.0	4.1	3.6	2.6
% Missing waist to height ratio	5.8	1.3	2.1	1.9	3.4
% Missing childhood social class	5.7				

Abbreviations: BMI, body mass index; WHtR, waist to height ratio
